# Microbial volatile organic compound emissions from *Stachybotrys chartarum* growing on gypsum wallboard and ceiling tile

**DOI:** 10.1186/1471-2180-13-283

**Published:** 2013-12-05

**Authors:** Doris A Betancourt, Ken Krebs, Scott A Moore, Shayna M Martin

**Affiliations:** 1 National Risk Management Research Laboratory, Air Pollution Prevention and Control Division, U.S. Environmental Protection Agency, E305-03, Durham, NC 27711, USA

**Keywords:** *Stachybotrys chartarum*, MVOC, Water-damaged building materials

## Abstract

**Background:**

*Stachybotrys chartarum* is a filamentous mold frequently identified among the mycobiota of water-damaged building materials. Growth of *S. chartarum* on suitable substrates and under favorable environmental conditions leads to the production of secondary metabolites such as mycotoxins and microbial volatile organic compounds (MVOCs). The aim of this study was to characterize MVOC emission profiles of seven toxigenic strains of *S. chartarum*, isolated from water-damaged buildings, in order to identify unique MVOCs generated during growth on gypsum wallboard and ceiling tile coupons. Inoculated coupons were incubated and monitored for emissions and growth using a closed glass environmental growth chamber maintained at a constant room temperature. Gas samples were collected from the headspace for three to four weeks using Tenax TA tubes.

**Results:**

Most of the MVOCs identified were alcohols, ketones, ethers and esters. The data showed that anisole (methoxybenzene) was emitted from all of the *S. chartarum* strains tested on both types of substrates. Maximum anisole concentration was detected after seven days of incubation.

**Conclusions:**

MVOCs are suitable markers for fungal identification because they easily diffuse through weak barriers like wallpaper, and could be used for early detection of mold growth in hidden cavities. This study identifies the production of anisole by seven toxigenic strains of *Stachybotrys chartarum* within a period of one week of growth on gypsum wallboard and ceiling tiles. These data could provide useful information for the future construction of a robust MVOC library for the early detection of this mold.

## Background

*Stachybotrys chartarum* is a filamentous mold usually identified among the mycobiota of wet-damaged, cellulose-containing building material [1,2]. *S. chartarum* is usually referred to as “toxic mold”; toxicity has been associated with exposure to spores and production of mycotoxins [3-5]. In addition, *S. chartarum* and other indoor molds have been linked to damp building-related illnesses (DBRI) such as allergic reactions of the upper respiratory system (e.g. irritated eyes, nose and throat) [6]. Likewise, cases of idiopathic pulmonary hemosiderosis have been associated with *S. chartarum* indoor exposures [7,8]. Also, *S. chartarum* may trigger immunologic, neurologic, and oncogenic disorders [5,7,9].

Proper risk management decisions are necessary whenever *S. chartarum* is identified in mold-infested environments for the proper remediation of this mold and minimal exposure of occupational workers to its toxic effects [10,11]. At present, there are no standardized protocols to identify the need for mold-remediation for indoor built environments. Most of the published mold-remediation guidelines recommend visual inspection for fungal growth as part of the assessment for mold-remediation at damp or water-damaged settings. Usually by the time visible mold growth is observed, it implies that inaccessible areas within the building construction are already mold-contaminated [11,12]. The implementation of new technologies for close monitoring of secluded, damp spaces is necessary for the early detection of mold growth. Several studies suggested the use of microbial volatile organic compound (MVOC) profiles as a diagnostic tool to determine mold-related problems in homes and buildings [13-15]. MVOCs are volatile organic chemical emissions associated with mold metabolism and may be linked to some of the adverse respiratory conditions generated by *S. chartarum*[16-19]. Combinations of MVOC emissions generate characteristic odors; these are detected prior to visual mold growth in buildings where occupants complaint of poor indoor air quality [20,21]. MVOCs are suitable markers because they easily diffuse through weak barriers like wallpaper and small crevices [12,15,20]. Likewise, they could be used for early detection of mold growth in hidden cavities (i.e. air ducts) and infrequently-visited places such as attics, crawl spaces and basements [12,22]. Several studies suggest that MVOC emission patterns could be used for the identification and classification of closely related microorganisms [23,24]. Larsen and Frisvad [25] analyzed the MVOCs emissions pattern of 47 *Penicillium* taxa and showed and the MVOCs emission profiles were unique enough to classify *Penicillium* to the species level. In a previous study, our laboratory characterized MVOCs emitted by three toxigenic strains of *S. chartarum* when grown on Sabouraud Dextrose Agar (SDA) and gypsum wallboard [26]. In the present study, we included seven toxigenic strains of *S. chartarum* to identify unique MVOCs for this mold to help in the construction of a robust MVOC library. An MVOC fingerprinting profile will be very useful for the implementation of a method for the early detection and identification of mold contamination when other signs of mold growth are absent. These studies are expected to advance our basic understanding of the physiology of *S. chartarum* and provide useful knowledge for the early detection and control of this toxigenic mold.

## Methods

### Test organisms

Spores from seven toxigenic strains of *Stachybotrys chartarum* were used in this study. Strains ATCC 201210, ATCC 208877, ATCC 62762, ATCC 46994, and ATCC 34916 were obtained from the American Type Culture Collection (Manassas, VA); and strains RTI 3559 and RTI 5802 were isolated from water-damaged homes and were obtained from the RTI International Collection (Research Triangle Park, NC). Prior to testing, all *S. chartarum* strains were grown on SDA (Sabouraud Dextrose Agar) and characterized microscopically to verify purity of the culture. Spore suspensions were prepared as described in Crow et al. [27] with modifications for harvesting mold spores [28]. All *S. chartarum* strains were individually grown on SDA plates until spore production was observed. Approximately 4–5 plates were grown for each strain. Spores were harvested from plates with 3 ml of 0.01 M phosphate buffer containing 0.05% (v/v) Tween 20 (Sigma Chemical, St Louis, MO, USA) at pH 7.0 (PBT pH 7.0) by gently scraping the surface of the plate with a sterile bent glass rod. The spore suspensions of the 4–5 plates were combined and centrifuged at 12,000 × g for 5 min. The supernatant was decanted leaving the spore pellet intact. The pellet was washed three times with 10 ml of the 0.01 M PBT and stored at 4°C until needed. Total spore count of the stock spore suspension was determined by direct microscopic counting using a hemocytometer. The spore suspension was examined microscopically to verify purity of the spores (i.e., absence of hyphae). When needed, this stock of spore suspension was diluted to the desired concentration (spores/ml) using 0.01 M PBT.

### Test substrates

Gypsum wallboard (W) and ceiling tiles (C) coupons were chosen as the cultivation substrate. The composition of the gypsum wallboard used was gypsum core (CaSO_4_ · 2H_2_O) wrapped with paper. The composition of ceiling tile was wood fiber (0-60%) and fibrous glass (0-13%). Both materials were purchased at local vendors. W and C were cut into 3 in. × 1.5 in. (7.62 cm × 3.81 cm) coupons. All substrates were individually steam – sterilized by autoclaving prior to inoculation. To provide a suitable moist condition for the germination of *S. chartarum* spores, sterile coupons were individually placed on a sterile glass Petri dish and wetted with 4 ml of sterile deionized H_2_O. Previous studies showed that *S. chartarum* grows on pre-wetted building materials at relative humidity below 100% [29]. All H_2_O was allowed to absorb prior to inoculation. Coupons were inoculated by pipetting 100 μl of spore suspension at the surface (usually with 10 spots of 10 μl arranged in an X configuration). The coupons’ preparation and the spiking procedure were performed in accordance with the ASTM guidelines D 6329–98 [30]. Spore concentration was 10^5^ – 10^6^ per coupon.

### Sampling for MVOC emissions from static test chambers

**Figure 1:**
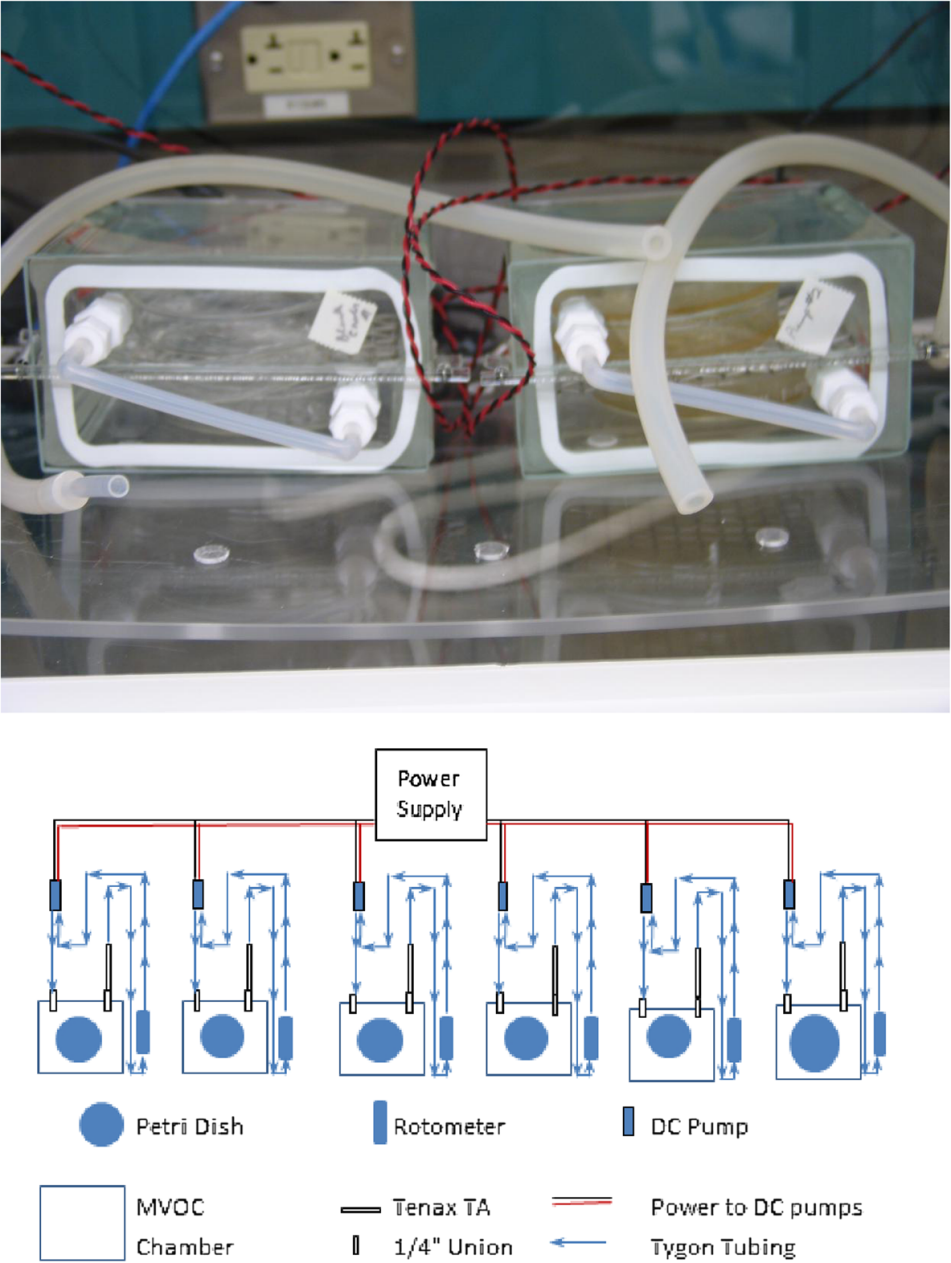
**Experimental setup.** The experimental setup allows for easily introducing the sorbent tubes into the sample loop without the need to open the growth chambers. A miniature pump draws the headspace from the chambers into the sorbent tube. The sample loop continues to a rotameter, where airflow is measured and is then transferred back into the growth chambers, thus providing a completely enclosed sample trajectory.

Figure 1 shows the experimental setup for the collection of MVOC emissions. Coupons inoculated with the predetermined spore load were contained in a static environmental growth chamber to quantitatively determine MVOC emissions. These chambers consisted of all-glass chambers, 4 ¾″ W × 2 ½″ D × 4 ½″ H (12 cm × 6.4 cm × 11.5 cm) (General Glassblowing Co., Inc., Richmond, CA) which were modified to include a face plate with two ¼″ Teflon bulkhead unions (with fritted glass disks); three glass culture plates (without lids), each with a test coupon; a wire mesh separator; 0 to 1 Lpm Gilmont flowmeter (Cole Palmer, Vernon Hills, IL) and an individual small sample pump. The size of each chamber was approximately 820 ml.

The testing period was 21 to 28 days of incubation at room temperature. Each experimental run included one or two strains of *S. chartarum* (each tested individually) and only one type of coupon. Each strain was tested in duplicate chambers. Each run included a control chamber with no coupons and a negative control consisting of a chamber with sterile, un-inoculated coupons. The MVOC sampling media were Supelco Tenax TA tubes (Sigma-Aldrich, St. Louis, MO). On day one, three spore-loaded coupons, each placed in a glass Petri dish, were introduced into each of the chambers. The control and test chambers were closed and allowed to equilibrate overnight at room temperature prior to the initiation of the testing period. After the equilibration period, the air from the headspace was collected onto Tenax TA tubes for 90 minutes at a nominal airflow of 0.05 liter per minute. Weekly headspace samples were collected within a period of 21 to 28 days.

MVOC samples collected on Tenax TA tubes were temperature desorbed according to published procedures described in EPA Method TO −17 and analyzed using an Agilent 6890/5973 Gas Chromatography/Mass Spectrometry (GC/MS) with Perkin Elmer Automated Thermal Desorber 400 system (PE ATD 400). For the instrument calibration, the relative response factor (RRF) method based on peak areas of extracted ion of target analytes relative to that of the internal standard was used. Gas phase d_8_-toluene was used as the internal standard. The following MVOCs, identified in a previous study, were used to calibrate the instrument: isoamyl alcohol (3-methyl-1-butanol), styrene, 3-octanone, anisole (methoxybenzene), cyclohexanol, and naphthalene, all purchased from Chem Service (West Chester, PA, USA); 4-methylanisole (1-methoxy-4-methylbenzene), 3-methylanisole (1-methoxy-3-methylbenzene), and 3,5-dimethoxytoluene purchased from Sigma-Aldrich (St. Louis, MO, USA) [26]. The calibration standards were prepared at five concentration levels ranging from approximately 4 to 400 ng/μl in CH_3_OH. Two μl of standards were spiked on each Tenax TA tube for the calibration. The practical quantification limit (PQL) which is the lowest calibration concentration was 8 ng/tube for each target analyte.

Target MVOC values in the samples are reported in micrograms per cubic meter (μg/m^3^). The MVOC concentration (C) was determined using Equation 1.

(1) C=M/V

Where: M is the mass of the MVOC measured on each Tenax sampling tube, ng; V is the air sample volume, liter; and C is the concentration, μg/m^3^.

Other fungal metabolites were identified with less certainty using a general mass spectral library available from the National Institute of Standards and Technology (NIST). VOC profiles were generated for each chamber. For each test period we had three types of VOC profiles: background VOCs; negative control VOCs; and positive controls VOCs. Background VOCs were those detected from the chambers without test coupons. Negative control VOCs were the emissions identified in chambers with test coupons without mold spores; most of the VOCs in these chambers were a combination of background and emissions from the wallboard (or ceiling tile) coupons. Positive control VOCs were those emitted from the coupons with mold spores; these emissions were a combination of MVOCs plus the previously mentioned VOCs. By comparing the three profiles, we identified the MVOCs emissions as *S. chartarum* grew either in W or C.

### Determination of mycotoxin and colony-forming unit (CFU)

Coupons loaded with *S. chartarum* spores were placed inside sterile glass Petri dishes and incubated in static growth chambers during the same testing period as the MVOC chambers. To verify the toxigenicity of the *S. chartarum* strains, we used the Envirologix QuantiTox kit for trichothecenes (Envirologix Inc., Portland, ME). The manufacturer’s protocol was used for mycotoxin extractions and assays. CFU analysis was done to monitor viability and growth of *S. chartarum* during the test period. The CFU analysis was done as described by Betancourt et al. [31].

## Results and discussion

In this study, we followed the MVOCs emissions from seven toxigenic strains of *S. chartarum* as they grew on cellulose-based gypsum wallboard (W) and ceiling tile (C). These essential building materials, used in the construction of walls and ceilings, are known to support microbial growth and become mold-colonized in a short period of time in damp or water-damaged indoor environments. Under these conditions, *Stachybotrys chartarum* is frequently identified among the mycobiota [1,2,32,33].

**Figure 2:**
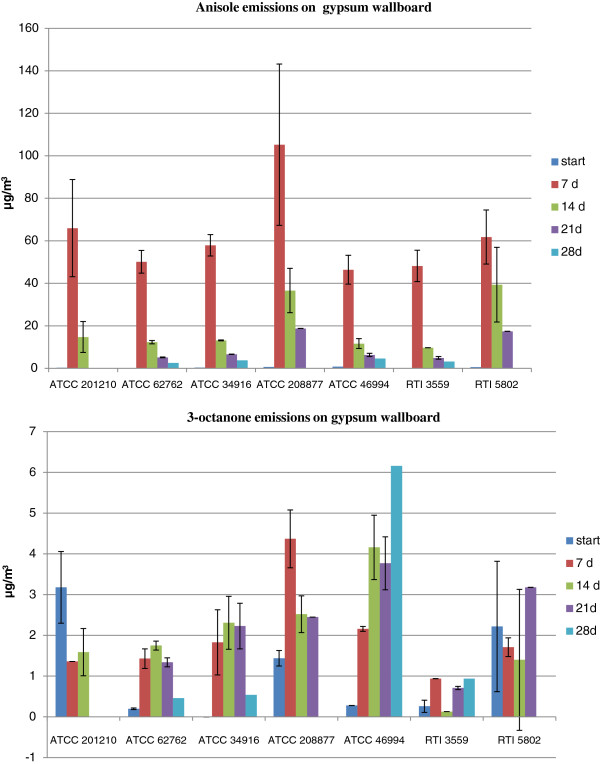
**Anisole and 3-octanone emissions on gypsum wallboard.** Anisole and 3-octanone emission was followed, as a function of time, during the growth of the different strains of *S. chartarum* on gypsum wallboard. The bar graph shows the mean ± SD of anisole and 3-octanone emissions.

**Figure 3:**
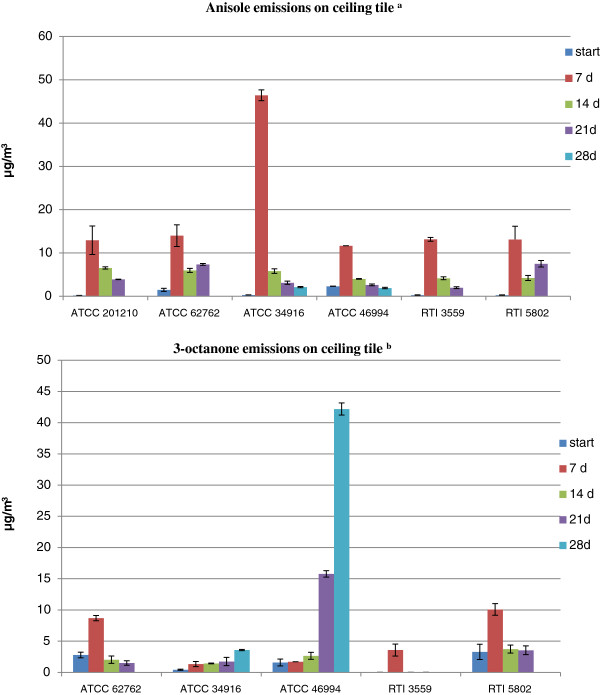
**Anisole and 3-octanone emissions on ceiling tile.** The bar graph shows the mean ± SD of anisole and 3-octanone emissions for six independent Sc strains growing on ceiling tile. ^a.^*S. chartarum* ATCC 208877 MVOCs emissions not tested on ceiling tile; ^b.^ 3-octanone emissions for *S. chartarum* ATCC 201210 below detection limit.

Additional file 1: Table S1 summarizes the MVOC emission profiles of *S. chartarum* growing on W and C. Most of the MVOCs identified were alcohols, ketones, hydrocarbons, ethers and esters. All these MVOCs have previously been reported as fungal metabolites [14,20,21,26,34-39]. The highlighted MVOCs were those emitted by four or more strains of *S. chartarum* on one or both of the substrates. These MVOCs were: anisole (methoxybenzene); 3-octanone; 3-methyl-3-buten-1-ol; 2-butanol; 2-(1-cyclopent-1-enyl-1-methylethyl) cyclopentanone; and 3,4-dihydro-8-hydroxy-3-methyl-(R)-1H-2-Benzopyran-1-one. Only the MVOCs emitted in both chambers (i.e., in duplicate) for the same mold strain were reported. Several studies showed that MVOC emissions’ profiles are very diverse; i.e., they vary depending on the fungi, the types of substrates available, and the existent environmental conditions (i.e., moisture, temperature) [14,40,41]. In this study, we observed this variability among the different *S. chartarum* strains and even within the same *S. chartarum* strain growing on different substrates (Additional file 1: Table S1). However, some MVOC emissions were highly reproducible even among different *S.chartarum* strains. We measured the MVOC concentrations of the following: anisole (methoxybenzene), 3-octanone, 3-methyl-1-butanol (isoamylalcohol), styrene, cyclohexanol, 4-methylanisole (1-methoxy-4-methylbenzene), 3-methylanisole (1-methoxy-3-methylbenzene), naphthalene, and 3,5-dimethoxytoluene based on the results of a previous study [26]. Only the concentrations of anisole and 3-octanone are reported; all the other MVOC tested were below detection limits (data not shown). Tables 1 and 2 summarize the concentrations of anisole (methoxybenzene), 3-octanone, mycotoxin and corresponding colony forming units (CFU) during different incubation times. Figures 2 and 3 represent the emissions pattern of both MVOCs on W and C, respectively. Our study showed that all seven strains (except ATCC 208877 which was not grown on C) emitted anisole on both wallboard and ceiling tile after 1 week of incubation and its concentration peaked within this timeframe. The concentration of anisole generated by the different strains was generally higher when grown on wallboard than on ceiling tiles (compare Figures 2 and 3 and note the difference in the scale of the Y-axis). Furthermore, the error bars were found to be larger for the gypsum wallboard (Figure 2) than those for ceiling tile (Figure 3); this is probably due to differences in the composition of the nutrient availability in the two building material as evident from the higher rate of anisole emission from the gypsum wallboard as compared to ceiling tile.

**Table 1:** **Growth, MVOC emissions and mycotoxin production by *Stachybotrys chartarum *** **growing on gypsum wallboard**

***Stachybotrys chartarum *** **strain**	**Substrate**^**a**^	**Incubation period**	**Anisole concentration**	**3-octanone concentration**	**Mycotoxin concentration**	**CFU log**_**10**_
		**(Days)**	**(μg/m**^**3**^**)**	**(μg/m**^**3**^**)**	**(ppb)**	**Mean ± SD**
			**Mean ± SD**^**b **^**(n)**^**c**^	**Mean ± SD (n)**		
ATCC 201210	W	Start	0.25 ± 0.05 (2)	3.18 ± 0.88 (2)	ND^d^	6.38 ± 6.44
		7 d	65.92 ± 22.87 (2)	1.36 (1)	ND	9.34 ± 8.99
		14 d	14.71 ± 7.27 (2)	1.59 ± 0.58 (2)	ND	9.96 ± 9.09
ATCC 62762	W	Start	0.12 ± 0.02 (2)	0.20 ± 0.02 (2)	0.2	6.1 ± 5.91
		7 d	50.1 ± 5.35 (2)	1.43 ± 0.24 (2)	< 0.2	6.59 ± 6.03
		14 d	12.26 ± 0.78 (2)	1.75 ± 0.11 (2)	0.2	7.31 ± 6.83
		21 d	5.10 ± 0.18 (2)	1.34 ± 0.11 (2)	2.0	6.90 ± 6.56
		28 d	2.52 (1)	0.46 (1)	> 18	8.25 ± 7.45
ATCC 34916	W	start	0.34 ± 0.12 (2)	BDL^e^	< 0.2	TFTC
		7 d	57.85 ± 5.03 (2)	1.83 ± 0.80 (2)	> 18	9.45 ± 8.48
		14 d	13.10 ± 0.21 (2)	2.31 ± 0.65 (2)	> 18	9.94 ± 9.31
		21 d	6.57 ± 0.08 (2)	2.23 ± 0.56 (2)	> 18	10.45 ± 9.95
		28 d	3.75 (1)	0.54 (1)	> 18	9.9 ± 9.19
ATCC 208877	W	Start	0.62 ± 0.09 (3)	1.44 ± 0.19 (2)	< 0.2	5
		7 d	105.19 ± 37.96 (3)	4.37 ± 0.71 (2)	0.2 < x < 2.0	7.99 ± 7.40
		14 d	36.58 ± 10.44 (2)	2.52 ± 0.45 (2)	18	9.55 ± 8.9
		21 d	18.72 (1)	2.45 (1)	2.0 < x < 18	9.49 ± 9.06
ATCC 46994	W	Start	0.75 ± 0.05 (2)	0.28 (1)	< 0.2	TFTC
		7 d	46.37 ± 6.78 (2)	2.16 ± 0.06 (2)	0.2	8.86 ± 8.83
		14 d	11.60 ± 2.31 (2)	4.16 ± 0.79 (2)	0.2 < x < 2.0	9.78 ± 9.30
		21 d	6.25 ± 0.76 (2)	3.77 ± 0.65 (2)	0.2 < x < 2.0	10.10 ± 9.52
		28 d	4.56 (1)	6.16 (1)	0.2 < x < 2.0	10.47 ± 9.32
RTI 3559	W	Start	0.15 ± 0.03 (2)	0.26 ±0.15 (2)	0.2	6.22 ± 5.61
		7 d	48.15 ± 7.39 (2)	0.94 (1)	18	8.96 ± 9.07
		14 d	9.64 (1)	0.13 (1)	18	10.36 ± 9.64
		21 d	4.89 ± 0.64 (2)	0.71 ± 0.04 (2)	18	10.29 ± 9.82
		28 d	3.16 (1)	0.94 (1)	> 18	9.27 ± 8.36
RTI 5802	W	Start	0.58 ± 0.11 (3)	2.22 ± 1.60 (2)	< 0.2	5.22 ± 4.76
		7 d	61.74 ± 12.72 (3)	1.71 ± 0.23 (2)	0.2	8.5 ± 7.53
		14 d	39.32 ± 17.57 (2)	1.40 ± 1.73 (2)	0.2	9.34 ± 8.99
		21 d	17.38 (1)	3.18 (1)	2.0	10.45 ± 9.40

^a^W, gypsum wallboard; ^b^SD, standard deviation; ^c^n, number of chambers with same strain, tested during same incubation period; ^d^ND, not determined; ^e^BDL, below detection limit.

**Table 2:** **Growth, MVOC emissions and mycotoxin production by *Stachybotrys chartarum *** **growing on ceiling tile**

***Stachybotrys chartarum *** **strain**	**Substrate**^**a**^	**Incubation period**	**Anisole concentration**	**3-octanone concentration**	**Mycotoxin concentration**	**CFU log**_**10**_
		**(Days)**	**(μg/m**^**3**^**)**	**(μg/m**^**3**^**)**	**(ppb)**	**Mean ± SD**
			**Mean ± SD**^**b **^**(n)**^**c**^	**Mean ± SD (n)**		
ATCC 201210	C	Start	0.15 (2)	BDL^e^	ND^d^	ND
		7 d	12.91 ± 3.29 (2)	BDL	ND	ND
		14 d	6.51 ± 0.26 (2)	BDL	ND	ND
		21 d	3.86 ± 0.05 (2)	BDL	ND	ND
ATCC 62762	C	Start	1.45 ± 0.35 (2)	2.77 ± 0.45 (2)	< 0.2	TFTC
		7 d	13.97 ± 2.50 (2)	8.68 ± 0.42 (2)	18	8.07 ± 7.55
		14 d	5.94 ± 0.47 (2)	2.02 ± 0.59 (2)	18	8.07 ± 7.55
		21 d	7.33 ± 0.21 (2)	1.49 ± 0.36 (2)	> 18	8.95 ± 8.74
ATCC 34916	C	Start	0.28 ± 0.01 (2)	0.40 ± 0.09 (2)	< 0.2	TFTC
		7 d	46.41 ± 1.25 (2)	1.32 ± 0.41 (2)	> 18	9.9 ± 9.19
		14 d	5.78 ± 0.53 (2)	1.42 ± 0.06 (2)	> 18	9.54 ± 9.05
		21 d	3.09 ± 0.37 (2)	1.73 ± 0.66 (2)	> 18	9.66 ± 9.22
		28 d	2.08 ± 0.14 (2)	3.56 ± 0.10 (2)	18	8.02 ± 8.00
ATCC 46994	C	Start	2.28 ± 0.02 (2)	1.57 ± 0.55 (2)	< 0.2	5.76 ± 5.91
		7 d	11.64 (1)	1.69 (1)	2.0	9.67 ± 9.11
		14 d	3.98 ± 0.08 (2)	2.64 ± 0.56 (2)	0.2 < x < 2.0	9.67 ± 9.26
		21 d	2.6 ± 0.2 (2)	15.76 ± 0.52 (2)	0.2 < x < 2.0	9.98 ± 9.52
		28 d	1.87 ± 0.16 (2)	42.18 ± 0.97 (2)	2.0	10.07 ± 9.38
RTI 3559	C	Start	0.22 ± 0.08 (2)	BDL	ND	ND
		7 d	13.12 ± 0.44 (2)	3.56 ± 0.96 (2)	ND	ND
		14 d	4.13 ± 0.33 (2)	BDL	ND	ND
		21 d	1.95 ± 0.21 (2)	BDL	ND	ND
RTI 5802	C	Start	0.23 ± 0.05 (2)	3.27 ± 1.22 (2)	< 0.2	TFTC
		7 d	13.10 ± 3.05 (2)	10.07 ± 0.93 (2)	2.0	9.06 ± 8.77
		14 d	4.19 ± 0.58 (2)	3.72 ± 0.64 (2)	0.2 < x < 2.0	9.06 ± 8.77
		21 d	7.48 ± 0.75 (2)	3.53 ± 0.70 (2)	2.0	9.53 ± 9.16

^a^C, ceiling tile; ^b^SD, standard deviation; ^c^n, number of chambers with same strain, tested during same incubation period; ^d^ND, not determined; ^e^BDL, below detection limit.

The highest concentration of anisole detected on wallboard was 105 ± 38 μg/m^3^ and on ceiling tile 46 ± 1 μg/m^3^. After two weeks of incubation, anisole concentration decreased and remained at detectable concentrations throughout the incubation period. The CFU and mycotoxin data clearly demonstrate that our experimental set-up supported spore production and mycotoxin synthesis (Tables 1 and 2). Previously, we reported similar results for anisole emissions using SDA and gypsum wallboard as growth substrates for *S. chartarum*[26]. Our results are in agreement with those reported by Wilkins et al. [42], Li [43] and Mason et al. [37]. All these studies reported anisole emissions as *S. chartarum* grew on gypsum wallboard [37,42,43] and cellulose insulation [43]. These studies also showed that anisole emissions are biogenic and are not commonly associated with general VOCs emitted from building materials. The aforementioned studies included *Aspergillus versicolor* and other indoor biocontaminants; anisole emissions were not detected among the MVOCs identified for all the molds tested on wallboard or any other building materials. Anisole has been proposed as a unique MVOC for *S. chartarum*[37]. However, in other studies, anisole emissions have been reported for *Aspergillus versicolor*[38,41,44]. As previously mentioned, these are instances that show the complexity of analyzing MVOC profiles due to the diversity of the environmental conditions, mold genera and substrate availability [34]. Our study showed that anisole emissions of *S. chartarum* are detectable within one week of incubation when growing on cellulose-containing building materials. We were able to demonstrate the reproducibility of anisole emissions for a total of nine *S. chartarum* strains (two from a previous study and seven new ones from the present study) during the first week of growth and the steady-state concentration maintained throughout the incubation period [26]. Robust MVOCs profiles with target compounds such as anisole might increase the sensitivity of a biosensor technology for the identification of *S. chartarum* in hidden cavities and spaces.

The other MVOCs frequently emitted by most of the *S. chartarum* strains tested was 3-octanone. The highest concentration on W was 4 ± 0.7 μg/m^3^ and on C was 42 ± 1 μg/m^3^. Emission patterns of this ketone were variable for both substrates. In ceiling tiles, the concentrations for several strains were below the detection limit. Previous studies reported 3-octanone as an MVOC derived from the degradation of fatty acids [25,42,45]. Several indoor fungi such as *Penicillium brevicompactum*, *Aspergillus versicolor*, *Eurotium amstelodami* and *Chaetomium globosum* among others emit this ketone as they actively grow on suitable building materials [46].

Gao et al. [36] studied the MVOC emissions of three toxigenic strains of *S. chartarum* when grown on rice and gypsum wallboard. We detected two MVOCs similar to those reported by Gao when *S. chartarum* was grown on W; these were: 2-(1-cyclopent-1-enyl-1-methylethyl) cyclopentanone and β-bisabolene. However, anisole and 3-octanone were not detected among the unique MVOCs reported by Gao et al. [36].

Mycotoxin assays showed that all the *S. chartarum* strains used in our investigation were toxigenic (Tables 1 and 2). Mycotoxin concentrations were variable among all the strains tested and were detected after seven days of incubation. Future studies will include HPLC analysis to identify the mycotoxins synthesized and molecular characterization of mycotoxins’ biosynthetic genes and sporulation genes to identify the possible association between anisole and other MVOC emissions and these cellular processes. Several studies suggested that high MVOC production might be associated with spore production and mycotoxin biosynthesis [20,47].

In the food industry, MVOCs have long been used as spoilage predictors for food and grains [48,49]. Karlshøj et al. [50] showed that certain types of MVOCs are emitted during mycotoxins biosynthesis. Therefore, recent trends are aimed at the development of electronic noses (e-noses) as indirect indicators of toxigenic fungi in food [50]. In indoor environments, the use of e-noses for the early detection of mold is a very promising technology. However, the interference of volatiles originating from building materials and the low concentrations of MVOCs are factors that need to be considered for the development of efficient sensors [51]. Schiffman et al. constructed an e-nose with a potential to effectively discriminate between several fungi species; and demonstrated that sensitive sensors are capable of discriminating specific volatiles [52]. We believe that the construction of a robust *Stachybotrys chartarum* MVOC library is the first step needed towards the development of an e-nose for the early detection of this mold in indoor environments. In this study (Additional file 1: Table S1), we provided the profiles of MVOCs from seven toxigenic strains of *S. chartarum* (in addition to the two strains we previously reported [26]) when grown on building materials that support mold growth under favorable conditions, and identified anisole (methoxybenzene) as a potential fingerprint for the early detection of this mold (Tables 1 and 2, and Figures 2 and 3). Indeed, the development of an e-nose for *S. chartarum* promises a major breakthrough for its e early detection in damaged indoor environments. Future studies will need to include the characterization and identification of the mycotoxins produced by *S. chartarum* in order to determine the correlation between toxigenic mycotoxin biosynthesis and MVOC emissions.

## Conclusions

Comparisons of MVOC emissions profiles of seven toxigenic strains of *S. chartarum* growing on gypsum wallboard and ceiling tile show that the ether (anisole) might be an excellent indicator for the growth and the presence of this mold in indoor environments. Robust MVOCs profiles with target compounds such as anisole might increase the sensitivity of a biosensor technology for the identification of *S. chartarum* in hidden cavities and spaces.

## Competing interests

The authors declare that they have no competing interests.

## Authors’ contributions

Conceived and designed the experimental protocols and performed static chambers tests: DAB, SAM. Coordinated the study, analyzed data, and wrote the manuscript: DAB. Performed all the GC-MS analysis: KK. Performed static chamber tests, mycotoxin assays and CFU: SMM. All authors read and approved the final manuscript.

## Supplementary Material

Additional file 1: Table S1 MVOC emissions of *Stachybotrys chartarum* growing on gypsum wallboard and ceiling tile. Click here for file
