# Supplementary material for: Microbial volatile organic compound emissions from Stachybotrys chartarum growing on gypsum wallboard and ceiling tile
Source: BMC Microbiol. 2013 Dec 5;13:283. doi: 10.1186/1471-2180-13-283 (PMC4234204; doi:10.1186/1471-2180-13-283)
Supplement: Additional file 1: Table S1 — MVOC emissions of Stachybotrys chartarum growing on gypsum wallboard and ceiling tile. [file 1471-2180-13-283-S1.doc]

**Table 1. MVOCs emissions of *Stachybotrys chartarum* growing on gypsum wallboard and ceiling tile**
